# Pityriasis lichenoides chronica in a patient on tafasitamab and lenalidomide therapy for diffuse large B-cell lymphoma

**DOI:** 10.1016/j.jdcr.2023.07.038

**Published:** 2023-08-12

**Authors:** Katie Roster, Rebecca Kann, Alina Zufall, Banu Farabi, Marian Russo, Kenneth Shulman, Bijan Safai

**Affiliations:** a New York Medical College, Valhalla, New York; b Dermatology Department, NYC Health + Hospital/Metropolitan, New York, New York; c Dermatology Department, NYC Health + Hospital/Coney Island, Brooklyn, New York

**Keywords:** cancer therapy, case report, pityriasis lichenoides chronica

## Introduction

Pityriasis lichenoides chronica (PLC) is a rare dermatosis that typically presents as recurrent erythematous papules, which can be asymptomatic or pruritic.^1^^,^^2^ Although the pathology of PLC is not well understood, it is thought to be an inflammatory reaction to antigenic trigger, which is mostly infectious in nature.^1^ More rarely, PLC may be seen as a paraneoplastic phenomenon or a reaction to a medication.^3^^,^^4^ Some researchers label PLC as a lymphoproliferative disorder given that these patients have demonstrated T-cell clonality, but this is highly debated in the literature.^1^^,^^2^^,^^5^ Herein, we present a case of PLC in a patient with diffuse large B-cell lymphoma (DLBCL) who developed a rash after initiating tafasitamab/lenalidomide therapy.

## Case report

**Fig 1:**
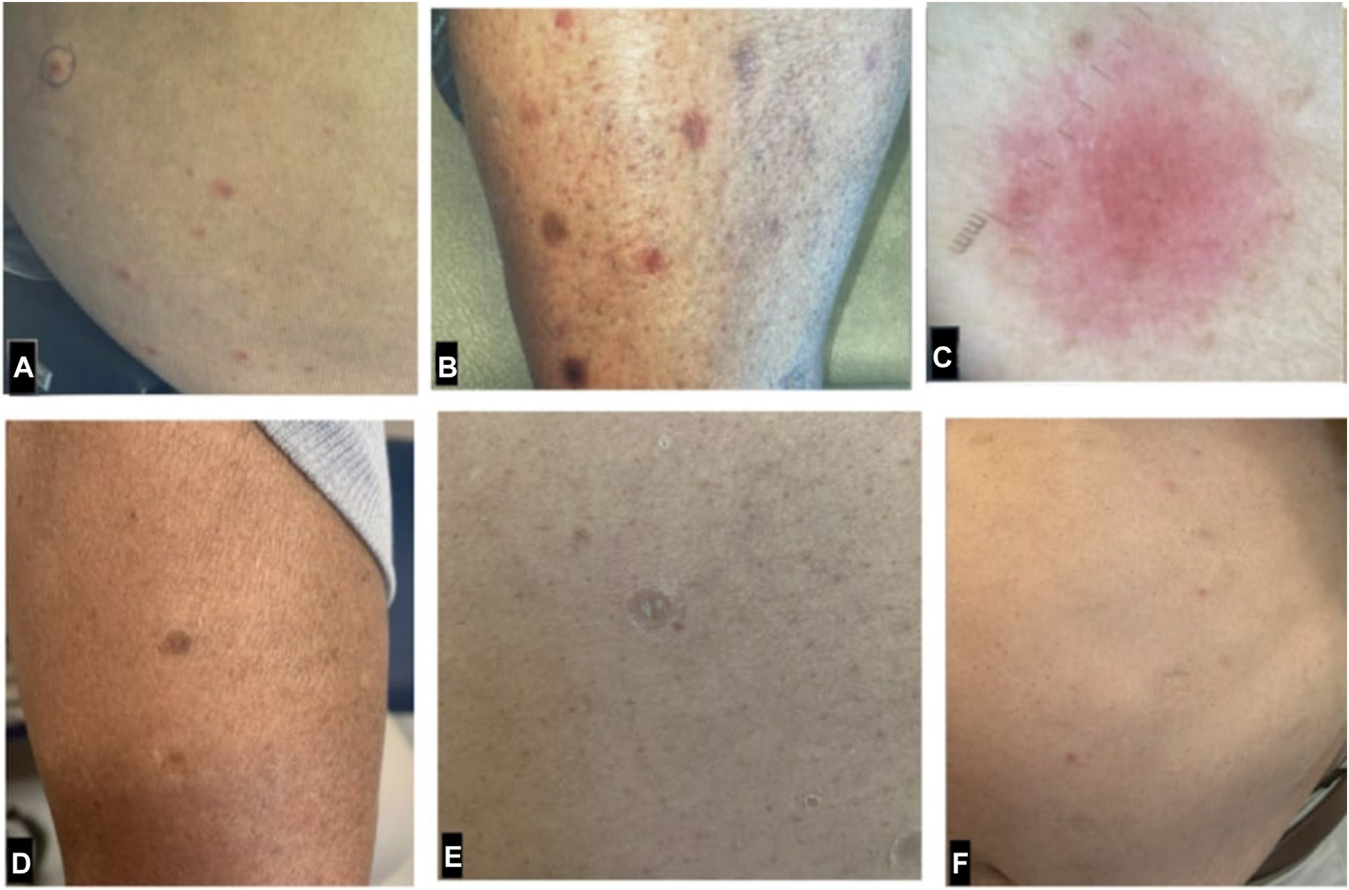
Numerous scaly and smooth, erythematous papules on the patient’s lower abdomen (**A**) and lower extremity (**B**). **C,** Dermatoscopy was nonspecific and showed red structureless areas. **D to F**, Papules at 1-month follow-up after treatment with betamethasone cream and cetirizine. **E,** A magnified view of a scaly, flat-topped, dull-pink papule at the 1-month follow-up.

A 69-year-old man with a diagnosis of DLBCL presented to our academic medical center with a pruritic rash that developed approximately 5 months after starting tafasitamab/lenalidomide therapy. At the time of initial evaluation, the rash had persisted for 3 weeks. On the fifteenth day of the patient’s sixth cycle of chemotherapy, due to the patient’s rash, the oncology department held the treatment until clearance was obtained from the dermatology department. According to the patient, his previous tafasitamab/lenalidomide treatment cycles had exacerbated the rash. However, the patient denied taking any new medications. His physical examination revealed multiple erythematous and hyperpigmented scaly papules and small plaques on the trunk and extremities (Fig 1, *A*,*B*). Dermatoscopy was nonspecific and revealed red structureless areas (Fig 1, *C*). Punch biopsies of the papules on the forearm and the right side of the abdomen were taken. The patient was prescribed betamethasone 0.05% cream twice daily and cetirizine 10 mg as needed for pruritus while waiting for the final pathology results.

**Fig 2:**
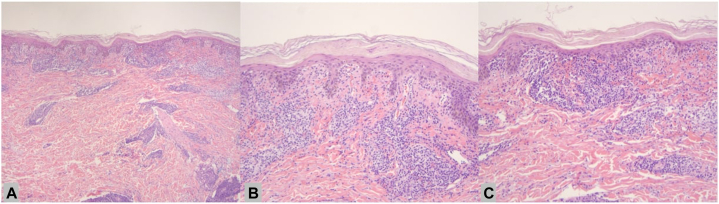
**A**, There is an interface dermatitis, superficial to mid-dermal, with parakeratosis (Hematoxylin-eosin stain; original magnification: ×4). **B, C,** Interface dermatitis with parakeratosis, basal vacuolization, a few individual necrotic keratinocytes, small perivascular lymphocytes, and extravasated erythrocytes in the papillary dermis (Hematoxylin-eosin stain; original magnification: ×10).

Histology revealed interface dermatitis with parakeratosis, basal vacuolization, and necrotic keratinocytes. Moreover, small perivascular lymphocytes and extravasated erythrocytes were noted in the papillary dermis (Fig 2, *A* to *C*). Immunostaining was positive for CD3^+^ T-cells, and there was no evidence of a B-cell infiltration or lymphomatoid papulosis, as indicated by the negative PAX5 and CD30 staining. There were no eosinophils on pathology. Together, these findings led to a diagnosis of PLC. Given the cyclic nature of the rash that flared after each chemotherapy cycle, PLC was thought to be secondary to tafasitamab/lenalidomide treatment.

Once a diagnosis was made, the patient was advised to continue using the betamethasone cream and cetirizine and continue chemotherapy. At the 1-month follow-up, the patient’s rash had significantly improved (Fig 1, *D* to *F*). The subsequent follow-ups did not reveal any recurrence of the rash.

## Discussion

In this case, we report the case of a 69-year-old man with DLBCL who presented with a pruritic rash after receiving his sixth cycle of tafasitamab/lenalidomide combination therapy. Tafasitamab is a CD19-directed cytolytic monoclonal antibody that is used in combination with lenalidomide, a thalidomide derivative with immunomodulating effects, including increased cytokine production.^6^

We suspect that the underlying pathology of the rash is consistent with an immune response in the setting of tafasitamab/lenalidomide therapy. Cutaneous adverse events are commonly reported in patients undergoing tafasitamab/lenalidomide combination therapy. In the open-label, single-arm, phase II L-MIND clinical trial, which evaluated the efficacy and safety of tafasitamab in combination with lenalidomide for patients with relapsed or refractory DLBCL, 37 (45.7%) out of 81 patients reported the occurrence of a rash. The most commonly reported rash morphologies were pruritus (*N =* 8, 9.9%) and rash not otherwise specified (*N =* 6, 7.4%).^7^ Although there have been no documented cases of PLC resulting from tafasitamab/lenalidomide, there have been several case reports of PLC from different medications, including chemotherapies.^5^ One case report presents the case of an 83-year-old woman who developed PLC after receiving an anti-CCR4 monoclonal antibody.^4^ Similar to our patient, she experienced symptoms months after initiating tafasitamab/lenalidomide therapy and her symptoms were exacerbated with each infusion.

Although research on the direct association between anti-CD19 medications and PLC is limited, it is reasonable to consider that a positive response to therapy would alter the B-cell response and impact T-cell activation. T-cell dysregulation has been implicated in the pathogenesis of PLC, supporting the plausibility of such a connection between our patient’s chemotherapy regimen and his dermatologic presentation.^2^

The histopathologic characteristics of PLC can vary, and it is challenging to differentiate between drug-related and non-drug-related PLC based solely on the histologic findings. A clinical histopathologic study that reviewed 10 cases of pityriasis lichenoides-like drug reaction reported a paucity of inflammatory cells, with eosinophils noted in 1 case.^4^ The histopathologic findings in our case align with the histologic characteristics of drug- and chemo-induced PLC, showing infiltration of lymphocytes in the papillary dermis and interface dermatitis with apoptotic parakeratosis, and a lack of eosinophils.

Moreover, previously documented cases have reported the development of PLC in the setting of a lymphoma and characterized the rash as a paraneoplastic skin disease rather than a drug reaction.^5^ This conclusion is somewhat controversial but is based on the evidence that the rash resolved with chemotherapy and treatment of the lymphoma. On the contrary, our patient experienced an exacerbation of symptoms with chemotherapy treatment.

## Conclusion

This case describes the development of PLC secondary to tafasitamab/lenalidomide chemotherapy treatment. Drug-induced PLC is not a contraindication to continue chemotherapy and can be managed conservatively with topical steroids and oral antihistamines.

## Conflicts of interest

None disclosed.

## References

[1] Schieke S.M., Wood G.S., Kang S., Amagai M., Bruckner A.L., Enk A.H., Margolis D.J., McMichael A.J., Orringer J.S. (2019). *Fitzpatrick’s Dermatology*.

[2] Bowers S., Warshaw E.M. (2006). Pityriasis lichenoides and its subtypes. J Am Acad Dermatol.

[3] Lu Y.Y., Liao J.B., Wu C.S., Hong C.H. (2014). Pityriasis lichenoids chronica as a paraneoplastic Dermatosis for primary splenic diffuse large B cell lymphoma. Indian J Hematol Blood Transfus.

[4] Magro C., Guo R., Nguyen G.H., Tsang H., Momtahen S. (2017). Pityriasis lichenoides-like drug reaction: A clinical histopathologic study of 10 cases. Dermatol Online J.

[5] Panizzon R.G., Speich R., Dazzi H. (1992). Atypical manifestations of pityriasis lichenoides chronica: development into paraneoplasia and non-Hodgkin lymphomas of the skin. Dermatology.

[6] Düll J., Topp M., Salles G. (2021). The use of tafasitamab in diffuse large B-cell lymphoma. Ther Adv Hematol.

[7] Salles G., Duell J., González Barca E. (2020). Tafasitamab plus lenalidomide in relapsed or refractory diffuse large B-cell lymphoma (L-MIND): A multicentre, prospective, single-arm, phase 2 study. Lancet Oncol.

